# A retrospective audit with subsequent cost and environmental analysis of a denosumab self-injection program

**DOI:** 10.1093/jbmrpl/ziae092

**Published:** 2024-07-17

**Authors:** Jack Boylan, Jane Turton, Zoe Hicks, Julia Cottam, Michael Stone

**Affiliations:** The Bone Research Unit, Llandough University Hospital, Llandough, Cardiff and Vale University Health Board, Wales, UK; The Finance Department, Cardiff and Vale University Health Board, Wales, United Kingdom; The Finance Department, Cardiff and Vale University Health Board, Wales, United Kingdom; The Bone Research Unit, Llandough University Hospital, Llandough, Cardiff and Vale University Health Board, Wales, UK

**Keywords:** aging, osteoporosis, diseases and disorders of/related to bone, health economics, epidemiology, antiresorptives, therapeutics, fracture prevention, practice/policy-related

## Abstract

The Metabolic Bone Health Department, Cardiff and Vale University Health Board, serves a local population of approximately 445 000 people. A retrospective audit of attendance data regarding the denosumab treatment clinic (the traditional treatment pathway) and the denosumab Self-Injection Program (SIP) was conducted to determine whether the SIP is both cost-effective and environmentally beneficial, compared to the traditional treatment pathway. Cost analysis was then conducted by the Finance Department. The audit was conducted over 3 years following the implementation of the service development; 233 patients had enrolled in the program at the time of the audit and 69 had completed 3 years of self-injected treatment. A control group of 497 patients were identified by the service. This group remained on the historical pathway and had consistent attendance activity over the 3-yr period from 2017 to 2019. Pre- and post-period activity of all patients on the program was compared, together with the activity for the independent control group. The SIP resulted in a reduction in clinical contacts, with financial analysis showing a total opportunity cost saving per patient of £420 per annum. There were obvious benefits to the patient of a reduced number of visits to a clinical site, which also resulted in an estimated carbon footprint reduction of 59 kg CO_2_ per patient per annum. The cost analysis is based on our organization’s 2022 charges. The SIP demonstrates that by focusing on care “closer to home”, it is possible to maximize resources, improve the patient experience through reduced travel, and reduce the environmental impact of healthcare.

## Introduction

Moving care “closer to home” has the potential to improve utilization of healthcare resources.^1^^,^^2^ In 2018, Cardiff and Vale University Health Board (CaV UHB) offered osteoporosis patients the opportunity to self-administer denosumab at home rather than attend the hospital outpatient clinic, through the Self-Injection Program (SIP). Patients who are offered the opportunity to self-administer their bone treatment are selected to ensure that they are clinically stable, physically able, and motivated to undertake the treatment in their own home.

A greater environmental focus is required not only globally but also at national and institutional level. In line with this, CaV UHB has developed a strategic plan to manage biodiversity across its sites.^3^ The health board has also set sustainability goals, which include reducing waste and enabling people to manage their health needs as close to home as possible.^4^

Our approach also aligns with national health policy in Wales. The concept of “prudent healthcare” was derived from the findings of the Bevan Commission^5^ and emphasizes the importance of effective use of resources alongside cost-effective prescribing and patient management. Prudent healthcare in turn forms a key part of the Welsh Government long-term plan for health and social care.^6^

In summary, this intervention is more than simply patient-centered; it aligns with organizational and political health objectives within Wales set at governmental level.

Denosumab is a monoclonal antibody that binds to the RANKL in the extracellular fluid and circulation. RANK signaling is an important mechanism in the regulation of osteoclast function and, crucially, bone resorption.^7^ Denosumab inhibits osteoclast formation, function, and survival,^8^ improving bone strength in patients with osteoporosis and reducing fracture risk. It is recognized that denosumab cessation leads to rapid reductions in bone mineral density (BMD), with bone turnover potentially elevating to above pre-treatment levels^9^ with an associated increased risk in vertebral fractures.^10^ As a result, patients usually require treatment over a number of years, with an indefinite cessation date.

## Background

### Original treatment pathway

**Figure 1:**
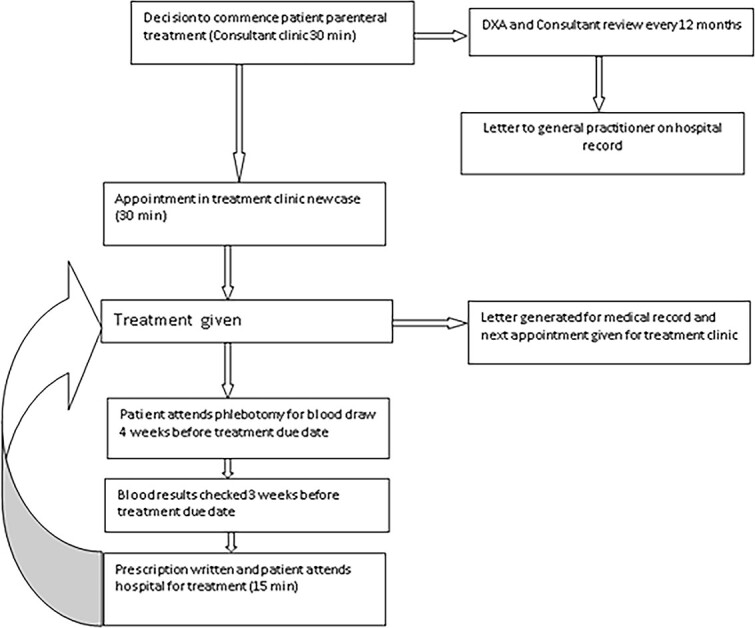
The original treatment pathway.

The historical patient pathway for treatment of osteoporosis with denosumab (Figure 1) included annual BMD assessment, an annual consultant clinic appointment, and twice-yearly administration of denosumab 60mg subcutaneously in a hospital outpatient clinic by specialist nurses. Blood tests to monitor renal function, calcium, and vitamin D levels are checked before each treatment; this is done either at the patient’s GP practice or the outpatient phlebotomy service, with the blood form generated by the Metabolic Bone Health Department. This service remains in place for those not in the SIP. The frequency of BMD and clinic appointment has changed for patients attending the outpatient service, these remain more frequent than the SIP and the National Osteoporosis Guideline Group^11^ would suggest, as these patients are not as clinically stable.

### SIP pathway

**Figure 2:**
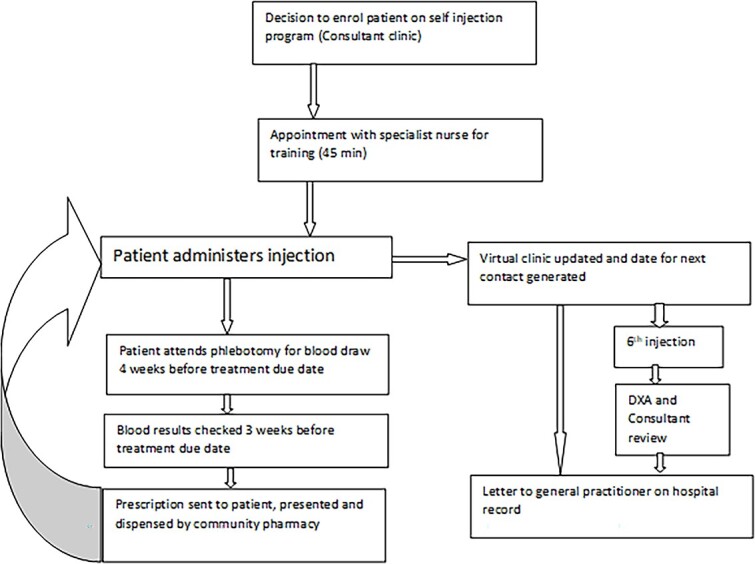
The Self-Injection Program (SIP) pathway.

Our main objective in designing the SIP was to improve patient convenience while maintaining an appropriate level of clinical supervision. In 2017, a small group of patients (*n* = 24) were invited to join the SIP. Feedback about the program was sought from this group by questionnaire. Twenty-nine percent were not interested, 10% asked for further information, 2% didn’t respond, and 59% were keen to take part. Having established that self-injection was acceptable and that there was interest in the opportunity to self-inject, more patients attending clinic for treatment were invited to join the program (Figure 2).

Prior to the first self-administered treatment, patients attend a nurse-led training session, which includes familiarization with denosumab. The process of self-injection is explained, and assessments including cognition and dexterity are undertaken. There is an opportunity to practice on a “dummy tummy” injection trainer. Finally, the program pathway is explained to the patient and written consent obtained. Patients receive a pack containing request forms for regular checks of serum calcium, vitamin D and renal function to ensure safe administration of treatment, a calendar of the dates of doses, and written instructions about self-injection.

When an injection is due, the patient attends for their blood test. The results are checked remotely and if they are in range, a prescription is sent to the patient and medication dispensed by a community pharmacy. After administering the injection, the patient notifies the SIP team.

Enrollment and self-administration are recorded using the diagnostics and therapeutics module of CaV UHB medical records “Patient Management System.”​ Prescribing is performed and recorded using the “Clinical Out-Patients Prescription,” and a date for the next patient contact is then generated. System memos are inserted into letters visible on Cardiff and Vale Clinical Portal and Welsh Clinical Portal (electronic systems within the health board and throughout the whole of Wales, respectively).

Theoretically, utilization of the SIP pathway should reduce the number of clinical contacts, as displayed in Table 1. Participants in the SIP contact the department twice a year to notify that an injection has been taken and are able to contact the clinical team if problems arise so that a medical consultation can be arranged. A full clinical review and BMD assessment is organized every 3 years.

**Table 1:** Activity over a 3-yr cycle: Historical hospital pathway versus New pathway prediction.

	**Activity over a 3-yr cycle**	
**Description**	**Historical hospital pathway**	**New pathway prediction**
Consultant outpatients	3	1
BMD scan	3	1
Treatment clinic	6	1^a^
Self-treatment	n/a	6

a Nurse-led training session.

### The retrospective audit

From the start of the audit period in July 2018 through to December 2020, the recruitment, training, and management of patients on the SIP were conducted by the department staff alongside pre-existing commitments. We recognized that this limited the number of patients offered the opportunity to self-inject. In 2020, AMGEN (UK) provided an unrestricted grant to employ a nurse for three days a week with responsibility for the SIP; this post became substantive within the NHS in December 2021.

The SIP was audited by the staff employed on the grant and the data used to inform our successful business case making our nursing post substantive within the organization. We then undertook the cost-benefit analysis as part of a “spread and scale” of the idea.

## Materials and methods

An independent cost-comparison analysis was conducted by CaVUHB finance department based upon data from a retrospective audit completed in July 2021. Patient appointment data from the bone treatment clinic (original treatment pathway) were used to identify the control group. The number of contacts with and attendance at the hospital for the control group was compared to the number of contacts with and attendance at the hospital of patients on the SIP. This utilized 2022 full costs for each health activity. Full costs reflect all CaVUHB expenditure allocated to activities and therefore overheads. Value Added Tax (VAT) in the UK in 2022 was 20%.

Accurate scrutiny was undertaken of all attendances pre- and post-enrollment using the first attendance to the self-injection clinic as the date of enrollment. To undertake a robust analysis of actual attendances, control comparisons were limited to patients who had been treated in the bone treatment clinic for 3 years, and in the SIP cohort, those who had completed 3 years post-enrollment.

### Theoretical SIP attendances and costs

At the time of the audit, 233 patients had been enrolled in the SIP between July 2018 and July 2021. This could deliver a saving of 233 Consultant Outpatient appointments and 233 corresponding DXA scans (based on those on the program for a year or more). In addition, there was a potential saving of 612 treatment clinic appointments (for patients on the program for more than 6 mo).

Using these audit data, initial theoretical savings were calculated. Table 2 identifies the cost of 233 patients on the hospital pathway as £871 653 over a 3-yr period; this compares to an equivalent cost of £398 430 for the new pathway. An opportunity cost saving of £473 233, equating to £2031 per patient over 3 years or £677 per patient per year.

**Table 2:** Activity over a 3-yr cycle: Opportunity cost savings.

		**Activity over a 3-yr cycle**		
	**Average unit cost**	**Hospital pathway**	**Self-injection pathway**	**Opportunity cost saving**
Consultant outpatients	£295	£885	£295	£590
BMD scan	£123	£369	£123	£246
Treatment clinics	£195	£1170	£195	£975
Denosumab	£183 (excl VAT)	£1318	£1098	£220
Average per patient cost over 3 years		£3742	£1711	£2031
**Average cost per year per patient**		£1247	£570	**£677**
**Total cost 233 patients over 3 years**		£871 653	£398 430	£473 223
**Annual equivalent 233 patients**		**£290 551**	**£132 810**	**£157 741**

Bold values draw attention to the opportunity cost saving per year per patient, as well as the annual cost for 233 patients in each pathway and the annual opportunity cost saving.

## Results

### Observed SIP attendances and costs

At the time of audit, 69 patients had completed 3 years of treatment pre- and post-enrollment and a total of 233 were enrolled and participating. Since the start of the program, 5 patients had chosen to revert to clinic attendance.

A control group of 497 patients were identified by the service. This group remained on the historical pathway and had consistent attendance activity over the 3-yr period from 2017 to 2019. The pre- and post-period activities were compared, together with the activity for the independent control group. Individual 2022 patient level costs were used for each attendance.

Table 3 contains theoretical and actual data for pre- and post-enrollment in the SIP program cohort of 69 patients, as well as the historical treatment pathway control group of 497 patients. For context, “treatment clinic” involved nurse-led administration of denosumab within the hospital.

**Table 3:** Activity over a 3-yr cycle: Theoretical attendances versus average observed attendances.

	**Activity over a 3-yr cycle**				
	**Theoretical attendances**		**Average observed attendances**		
	**SIP pre-enrollment**	**SIP post-enrollment**	**SIP pre-enrollment (*n* = 69)**	**SIP post-enrollment (*n* = 69)**	**Control group (*n* = 497)**
Consultant outpatients	3	1	3	0.9	2.3
BMD scan	3	1	1.9	0.8	1.6
Treatment clinic	6	1	6.7	2.2	5.2
**Total**	**12**	**3**	**11.6**	**3.9**	**9.2**
**Reduction in attendances if adopted self-injection pathway**			**7.7 (11.6–3.9)**	**n/a**	**5.3 (9.2–3.9)**

Bold values draw attention to the total’s of each column and to the reduction in attendances if adopted self-injection pathway.

Consistent with expectations, the number of appointments and hospital attendance reduced significantly. Consultant outpatient contacts were reduced by 70%, there was a 57.9% reduction in the number of DXA scans conducted and a 67.2% decrease in treatment clinic attendances. The total number of actual appointments reduced substantially pre- to post-enrollment, decreasing by 7.7 (11.6–3.9) per 3-yr cycle or 2.56 per annum.

Differences were discovered when comparing the theoretical and observed attendances. In the pre-enrollment group, the number of DXA appointments was fewer than expected (3 vs 1.9), while there were more treatment clinic attendances (6 vs 6.7). In the post-enrollment cohort, attendances were higher than expected at treatment clinics (2.2 vs 1). Finally, the control group had, on average, fewer appointments in every category of both theoretical and observed attendances than the pre-enrollment SIP cohort.

Table 4 details the total costs of each cohort, as well as an individual patient cost. For clarity, VAT is a tax that consumers pay when they buy goods or services in the UK, and is added to the price of a product at each stage of the supply chain, minus costs that have already been taxed. VAT is charged on medications dispensed and administered within a hospital setting, but not when medications are dispensed and administered in primary or community care setting. Therefore, an additional cost of £55 per patient per treatment is incurred in the historic pathway when compared to the SIP.

**Table 4:** 3-yr average cost of observed attendances versus average annual cost of observed attendances.

	**3**-**yr average cost of observed attendances**			**Average annual cost of observed attendances**		
	**SIP pre-enrollment**	**SIP post-enrollment**	**Control group**	**SIP pre-enrollment (*n* = 69)**	**SIP post-enrollment (*n* = 69)**	**Control group (*n* = 497)**
Consultant outpatients	£885	£266	£679	£295	£89	£226
BMD scan	£234	£98	£197	£78	£33	£66
Treatment clinic	£1307	£429	£1014	£436	£143	£338
Additional VAT—Prescribed in Hospital setting	£165		£165	£55		£55
**Total**	**£2591**	**£793**	**£2055**	**£864**	**£265**	**£685**
**Opportunity cost saving per patient per year**					**£599**	**£420**

Bold values draw attention to the total of each column and the opportunity cost saving per patient per year.

There was an annual cost of £864 per patient in the SIP cohort pre-enrollment and £265 post-enrollment. This represents an opportunity cost saving of £599 per patient per year. It should be noted that the opportunity cost saving is 11.53% lower than when compared to the theoretical pathway where savings of £677 per patients were identified. The variance reflects the difference in actual attendances when compared to the expected pathway attendances. The average cost of the control group was £685 per participant per year, which is lower than the SIP cohort pre-enrollment. When the post-enrollment costs are compared to the control group, the opportunity cost saving reduces to £420 per patient.

### The environmental impact of the SIP

At the time of cost and environmental analysis, the UK Government Sustainability Development Unit identified specific carbon equivalent emissions produced as a result of healthcare activities. These represented industry standard figures and can be used to calculate the impact on carbon foot printing of service redesign. An acute sector Outpatient Appointment was noted to produce on average 23 kg CO_2_e.

The average reduction in number of attendances identified in the pre- and post-enrollment cohort of 69 patients (see Table 3) is 7.7, over a 3-yr period, or 2.56 per annum. Therefore, each patient has saved on average 59 kg CO_2_e per year (2.56 × 23 kg CO_2_e). Through utilizing the SIP a total saving of 4071 kg CO_2_e per annum and 40917 kg CO_2_ over the 3-yr period for the entire cohort was estimated to be achieved.

## Discussion

The SIP program was developed as a clinical service because we were unable to share the ongoing management of patients with denosumab with primary care, and as a consequence the waiting time to initiate therapy for new patients was not clinically acceptable. The patient participants in our pilot group helped us to formulate a safe and acceptable program, balancing secondary care clinical supervision with patient convenience. We have not yet undertaken a formal re-evaluation of patient preference and satisfaction; however, our informal feedback is very positive.

During the audit period, the COVID pandemic occurred, causing widespread disruption to healthcare providers across the globe. We were able to sustain the SIP during this time and noticed an increased interest in the SIP as patients were keen to avoid hospital or primary care-based healthcare contact. We have not formally studied the DXA and fracture outcomes comparing patients attending the clinic for treatment with the SIP patients; however, as adherence to treatment is monitored, we do not expect there to be any difference.

The cost analysis of the SIP within CaV UHB demonstrates that by moving away from traditional methods of delivering care, it is possible to improve the patient journey and experience. NHS resources are under ever increasing pressure and scrutiny, across not only Wales but the whole of the UK. By utilizing the principle of providing care closer to home, we have demonstrated that there is a significant financial cost-saving opportunity while continuing to provide high-quality care.

We observed an additional benefit of the SIP program. Previously, patients within the department had annual bone density scans to monitor treatment response. Our experience of the SIP has resulted in a gradual change in practice, with a move away from annual scanning for those remaining on the traditional treatment pathway, likely resulting in further cost savings.

As previously described, our organization continues to place significant emphasis on ensuring healthcare provision incorporates the principles of sustainability. Not only does this impact the aforementioned opportunities for effective use of resources, it also links directly with the Health Board’s targets for environmental conservation. As demonstrated, the SIP results in a notable reduction in carbon footprint through minimizing patient travel to and from hospital.

The analysis is based on our organization’s 2022 cost quotations. We recognize that given the economic impact of the COVID pandemic and global economic turbulence (with significant fluctuations in inflation), these costs may have changed, limiting the extent to which we can extrapolate the data. Therefore, it is reasonable to assume that inflation, accompanied by subsequent monetary policy interest rate rises, will only have increased costs to healthcare services.

## Conclusion

Healthcare organizations within the National Health Service are under increasing pressure to use resources effectively, provide care closer to home and at the same time, reduce their environmental burden. With current fiscal constraints, this can often feel like an insurmountable task. Through the SIP, CaV UHB has demonstrated that it is possible to achieve all three of these aims with a single intervention, saving both money, patient time, and carbon impact.

Moving forward, the Metabolic Bone Health Department plans to expand the SIP to more patients interested in taking increased autonomy of their osteoporosis treatment. It is also hoped that the structure of this innovation could be utilized within other departments in our organization. In the wider NHS, we hope that this model of care could be utilized by other centers managing imminent fracture risk.

## Data Availability

The data that support the findings of this audit are available from the corresponding authors (Jack Boylan or Jane Turton).
